# Book Reviews

**Published:** 1827-12

**Authors:** 


### CRITICAL ANALYSES.

Quæ laudanda forent, et quæ culpanda, vicissim

Illa, prius, cretâ; mox hæc, carbone, *notamus.*—Persius.

*Reports of Medical Cases, selected with a view of illustrating the Symptoms and Cure of Diseases by a reference to Morbid Anatomy.* By Richard Bright, M.D. F.R.S. &c. Lecturer on the Practice of Medicine, and one of the Physicians to Guy's Hospital.—4to. pp. 231. Longman and Co. London, 1827.

The principal object of this work is to illustrate some of the appearances observable on the examination of diseases terminating in dropsical effusion. These are various, and some of them well known,—thus we are accustomed to acknowledge the connexion between certain diseases of the liver, the heart, and the lungs, and serous effusion into the various cavities. Obstructed circulation seems to be the common principle on which all these act: heart affections frequently throw the blood back, or more properly speaking, delay it in the venous system;—hepatic disease prevents the free circulation through the vena portæ; simple tumors, even if unconnected with disease, as pregnancy, by mere mechanical influence produce anasarcous accumulations. Another set of causes, probably operating in a different manner, are to be found in certain changes which the serous membranes undergo. But, besides all these, Dr. Bright is of opinion, that the kidneys are frequently diseased in dropsy; and this, not as an adventitious circumstance, but as constituting an important part in the pathological history of these effusions. Where these conditions of the kidney have occurred, the dropsy has frequently been found connected with the secretion of urine, containing albumen, and more or less coagulable on the application of heat; and it is further worthy of remark, that in these cases the liver has generally been found comparatively healthy: on the other hand, where the dropsy has been dependent on organic disease of the liver, the urine, as a general rule, has not coagulated by heat, nor have the kidneys been found diseased. "I have never yet (says Dr. Bright,) examined the body of a patient dying with dropsy, attended with coagulable urine, in whom some obvious derangement was not discovered in the kidneys:" whether these changes of structure are to be considered as having, in their early stages, given rise to an alteration in the secreting power, or whether the organic change be the consequence of a long continued morbid action, is doubtful; but our author thinks it probable that the change in the action of the kidney results from the various injurious causes operating upon that organ, through the medium of the stomach, deranging the balance of the circulation in the part, or perhaps producing actual inflammation.

Dropsy, from exposure to cold, is generally attended with albuminous urine, and Dr. Bright further points out the tendency which the kidneys have, under such circumstances, to throw off the red particles of the blood, producing various degrees of hæmaturia. In a form of dropsy very opposite to this, that, namely, which occurs in worn out and cachectic habits, the same phenomenon presents itself. But in all such cases as already stated, our author thinks the kidneys have been deranged either functionally or organically, or both.

Now these various positions which we have thus briefly laid down are exemplified by the detail of numerous cases, which are given at full length, and each of which is followed by the remarks which it has suggested to the author; after which, as a summing up of the evidence, there are some observations upon the whole generally. To follow so many cases through daily reports, would be tedious, nay, with our confined limits, impossible; but, as this is the part of the work having most claims to originality, and as the subject is full of interest, we have condensed somewhat Dr. Bright's observations, and present them to our readers as a general analysis of his views on this subject.

*Some general Remarks on the foregoing Cases.*

"From the observations which I have made, I have been led to believe that there may be several forms of disease to which the kidney becomes liable in the progress of dropsical affection: I have even thought that the organic derangements which have already presented themselves to my notice, will authorise the establishment of three varieties, if not of three completely separate forms, of diseased structure, generally attended by a decidedly albumious character of the urine.—In the *first*, a state of degeneracy seems to exist, which from its appearance might be regarded as marking little more than simple debility of the organ. In this case the kidney loses its usual firmness, becomes of a yellow mottled appearance externally; and when a section is made, nearly the same yellow colour slightly tinged with gray is seen to pervade the whole of the cortical part, and the tubular portions are of a lighter colour than natural. The size of the kidney is not materially altered, nor is there any obvious morbid deposit to be discovered. This state of the organ is sometimes connected with a cachetic condition of body, attended with chronic disease, where no dropsical effusion has taken place either into the cellular membrane or into the cavities of the body; I have found it in a case of diarrhœa and phthisis, and in a case of ovarian tumor. In the former it was connected with slight and almost doubtful coagulation of the urine by heat; in the latter I had omitted to examine the state of the urine. I also met with nearly the same condition of the kidney, with some opake yellow deposits interspersed through the structure, in the case of a man who died exhausted with diarrhœa brought on by hardships and intemperance, and in whose case the secretion of urine was very deficient, but whether coagulable or not I had no opportunity of ascertaining. When this disease has gone to its utmost, it has appeared to terminate by producing a more decided alteration in the structure; some portions becoming consolidated, so as to admit of very partial circulation; in which state the surface has assumed a somewhat tuberculated appearance, the gentle projections of which were paler than the rest, and scarcely received any of the injection which was thrown in by the arteries. In this more advanced stage, if it be the same disease, dropsy has existed, and the urine has been coagulable. (Sallaway, Case III.)

"The *second* form of diseased kidney is one in which the whole cortical part is converted into a granulated texture, and where there appears to be a copious morbid interstitial deposit of an opake white substance. This in its earliest stage produces externally, when the tunic is taken off, only an increase of the natural fine mottled appearance given by the healthy structure of the kidney; or under particular circumstances, gives the appearance of fine grains of sand sprinkled more abundantly on some parts than others. On making a longitudinal section, a slight appearance of the same kind is discovered internally, and the kidney is generally rather deficient in its natural firmness. After the disease has continued for some time, the deposited matter becomes more abundant, and is seen in innumerable specks of no definite form thickly strewed on the surface; and on cutting into the kidney these specks are found distributed in a more or less regular manner throughout the whole cortical substance, no longer presenting a doubtful appearance, but most manifest to the eye without any preparation; and other cases less advanced, requiring maceration in simple spring water for a few days to render them more obvious. When this disease has gone on for a very considerable time, the granulated texture begins to show itself externally, in frequent slight uneven projections on the surface of the kidney; so that the morbid state is readily perceived even before the tunic is removed. The kidney is generally rather larger than natural; sometimes it is increased very much, but at other times it is little above the natural dimensions.

"The *third* form of disease is where the kidney is quite rough and scabrous to the touch externally, and is seen to rise in numerous projections not much exceeding a large pin's head, yellow, red, and purplish. The form of the kidney is often inclined to be lobulated, the feel is hard, and on making an incision the texture is found approaching to semicartilaginous firmness, giving great redistance to the knife. The tubular portions are observed to be drawn near to the surface of the kidney : it appears, in short, like a contraction of every part of the organ, with less interstitial deposit than in the last variety. This form of disease existed in a case from which I had a drawing executed about three years ago, it also existed in Bonham (Case VIII.); and a most decidedly-marked instance of it may be found in Stewart where however the kidney was of a lighter colour than in the other cases, which were more of a purplish gray tinge. I believe the case of Smith belonged to the same. In most of these cases the urine has been highly coagulable by heat, at times forming a large curdled deposit, though in one case (Castles) where an approach to this appearance was found on the outside of the kidney, but with marked structural change in the liver, and with confirmed bronchial congestion, only a dense bran-like deposit of a brown colour was produced by the application of heat.

" Although I hazard a conjecture as to the existence of these three different forms of disease, I am by no means confident of the correctness of this view.

" Besides these three forms of disease, passing almost into each other and usually attended with decidedly coagulable urine, there are two other deranged conditions of the kidneys in which the coagulation is sometimes observable, but in a very subordinate degree, and often though observable on one day is quite lost on another. One of these morbid states consists in a preternatural softness of the organ ; the other in the blocking up of the tubular structure by small portions of a white deposit bearing the appearance of small concretions. In the former a corresponding loss of firmness has been observed in the structure of the liver, and the spleen and the parieties of the heart, the action of which organ had been observed during life to be deficient in force. In the other cases, besides the obstructed state of the uriniferous tubes, the whole structure of the kidney has been somewhat deranged, the cortical portion firmer than natural, and the tubular part has lost the regular convergency of the vessels, so that they have assumed a waved direction.—It is by no means improbable that we shall hereafter find many other sources of renal irritation to be connected with an analogous state of the urine.

*Observations on the Treatment.*

" In the foregoing statements it has been my great object to establish the fact, that certain dropsical affections depend more on the derangement of the kidneys themselves than has generally been supposed ; and that the albuminous nature of the urine frequently points out the particular cases in which these organs are the seat of disease. I wish that I were now able to add any thing completely satisfactory to myself with regard to the mode of treating these diseases of the kidney. It will be very obvious from a review of the cases I have cited, that they sometimes present difficulties so formidable as to defy the ordinary means of cure; indeed I am inclined to doubt whether it be possible, after the decided organic change has taken a firm hold on the kidney, to effect a cure, or even to give such relief as may enable the patient to pursue for a few years the occupations of life ; where, however, the mischief is less rooted, we may undoubtedly do much. In the treatment of the disease, as it occurs in sudden attacks of anasarca from intemperance and exposure, in its early stages, and before organic changes have taken place, we have two distinct indications to fulfil ;—we have to restore the healthy action of the kidney, and we have to guard continually against those dangerous secondary consequences which may destroy the patient at any period of the disease.

"The two great sources of casual danger will be found in inflammatory affections, more particularly of the serous, sometimes of the mucous membranes, and in the effusion of blood or serum into the brain, and the consequent occurrence of apoplexy. Of these secondary or casual dangers we have illustrative examples in many of the cases which have been stated above. Out of seventeen dissections, we have found ten or eleven betraying inflammation of the pleura, generally old, but sometimes of more recent date. We have found three instances in which the patients had suffered decided attacks of inflammation in the pericardium shortly before death; and in two of these cases we had proof of some previous affection of the same kind. In one only were the signs of inflammation in the peritoneum well marked. Five out of the seventeen had altogether escaped inflammatory affections of the serous membranes ; and one of these died with inflammation of the epiglottis. Thus then we have proof of the frequency of these attacks ; and at the same time it is obvious that they form no essential part of the disease, since in several of the best-marked cases there has been no reason whatsoever to suspect inflammation during life, and no traces of its existence have been discovered after death. With regard to the cerebral affections coming on in the progress of these diseases, we find in the cases above related both apoplexy and epilepsy to have occurred ; and a very well marked instance of the former was witnessed in a patient of the name of Macguire, in the Clinical ward in 1825.

"Purgatives generally act well ; the Elaterium in the case of Evans evidently gave much relief ; and all the saline laxatives which unite a certain degree of diuretic power are decidedly useful. Amongst these I have found the Supertartrate of Potash the most efficacious ; and the best mode of exhibiting it, when the stomach will admit, is by directing the patient to take a large draught of a mixture containing more of the salt than the water will dissolve, the first thing in the morning : and it will be seen that in some cases I have almost trusted entirely to this remedy. Where the stomach will not bear this mode of administering purgatives, the combination of Jalap, Supertartrate of Potash, and a little Ginger repeated from time to time, answers well, or even frequent doses of Castor Oil have been very useful.

"The diuretic remedy which I have generally used, has been the Squill in its different forms: but it has always acted best when given in combination with Hyoscyamus, or when a grain of Opium has been prescribed once or twice a day. Indeed I cannot but consider this an important part of the treatment, with a view to diminish the irritation of the kidneys, as well as to allay the general disturbance which must necessarily result to the constitution, from the circulation of blood which has been so imperfectly acted upon by these organs. Digitalis has in some instances been cautiously administered with temporary advantage, and seems by its power of checking the circulation to be well adapted to those cases where the pulse is sharp, as frequently occurs throughout the whole progress of this disease.

"One of the most important questions in the treatment of this class of dropsies, is the propriety of employing Mercury. It is consistent with the most successful treatment of many forms of inflammatory disease, that we should have recourse to the valuable combination of Calomel with Opium; and it is consistent with what is generally deemed good practice, that by the cautious use of mercury we should endeavour to produce more healthy action, and to promote absorption when there is reason to believe that disease has left any chronic morbid action tending to produce unhealthy deposit in glandular structures. Still however, the cases which have proved most successful in my own practice, have generally been those in which I have rigidly abstained from the use of mercury. In some cases I have seen the good effects of other remedies entirely interrupted by the mercurial action; and I have likewise seen several instances in which the cure, when mercurials have formed part of the plan, has been protracted to a great length; and a great many in which the full action of mercury has not prevented the regular progress of the disease, and its fatal termination. Yet I have undoubtedly seen well-marked cases of this disease with decidedly coagulable urine, when taken early, in which the free use of mercury to complete ptyalism has not prevented the patients from deriving great, perhaps even perfect relief, from the remedies with which it was combined,—these remedies having been bleeding, purging, and diuretics. Independently of the very great doubt which exists as to the advantage to be derived from mercury, there is one circumstance which most materially limits our power of employing it, and that is the violence and rapidity with which the ptyalism often comes on, and the great difficulty which is frequently experienced in restraining its effects: for when the cellular membrane is in the peculiar state of anasarca induced by this disease, the gums and cheeks are not capable of supporting the process of ulceration, and often pass into a state of gangrene.

"Where, as in a case to which I have only referred, we have a flaccid, watery and dissolved state of the kidney, I can point out no diagnostic symptoms by which it can be discovered, except such as show general debility of circulation and feebleness in the structure of the heart; for probably the feeble condition of the two organs may often be found coexistent. If this be the case, it is not improbable that Tonics will be the most appropriate remedies. In one or two cases of anasarca which I have lately had under my care, where from the feeble but extensive beat of the heart I was led to suppose that a feeble state of that organ existed, a combination of Sulphate of Quinine with Squill, effectually restored the patient. And occasionally we find anasarca even with coagulable urine so marked by debility, that tonics and steel give decided relief; probably it is as a tonic that the Uva Ursi is sometimes useful." (P. 67.)

Dr. Bright, however, in maintaining that the kidney is often at fault in cases of dropsy where the liver has been inflamed, by no means goes so far as to assert that the state of the latter may not likewise be the frequent cause of effusion, even where the kidneys are quite healthy. These morbid conditions of the liver are very various; but the most interesting, in a pathological point of view, is that in which the viscus was contracted, and throughout of a morbid structure, apparently by the deposition of minute portions of yellow matter, the surface covered by a very fine peritoneum, quite transparent, even more thin than usual, presented a general rough granular, and therefore uneven, surface, of what might be called liver-coloured red and yellowish gray. On being cut into, the same structure of a less red colour pervaded the whole. The liver was thicker and rounder than natural, and rather smaller, and on pressure broke down easily with a brittle or crisp fracture, uneven and granular. The gall-bladder, opake and thick, contained the usual quantity of bile; the common duct was pervious, but at its entry into the duodenum was contracted in a nipple-like projection, with an orifice not much larger than to admit the point of a pin. On opening the gall-bladder and letting out the deep-coloured viscid bile with which it was filled, a number of small yellow bodies, larger than millet seeds and soft, adhered to the villous surface of the gall-bladder, chiefly on the side where it is attached to the liver." The appearances in this case led Dr. Bright to suspect that part of the structural change depended on some deposit from the bile, the small white bodies found in the gall-bladder having a certain resemblance to the whiter parts of the liver. With a view of ascertaining this point, he transmitted a portion of diseased structure to Dr. Bostock, who obtained a quantity of what appears to have been *cholesterine;* but as the subject is new and interesting, we shall subjoin his letter.

*Upper Bedford Place, December 26th,* 1826.

Dear Sir,

"At your request I send you an account of some of the experiments which I made upon the portion of liver of Willoughby Taylor which you sent me in January last. It differed in its external characters from the ordinary aspect of this organ; it had a marbled appearance, and upon being closely examined, was found to consist of a number of small white granular masses, from the size of a pea to that of the most minute particle, imbedded in a basis of reddish brown substance. When a thin layer of the liver was viewed in the microscope, the distinction of the two parts was still more evident, the white masses being considerably translucent, while the connecting substance was perfectly opake.

"1. A portion of the liver was divided into thin slices, which were macerated in cold water, the water being changed daily. After this operation had been continued for three weeks, the red connecting part was evidently broken down and partially removed, while the white masses did not appear to be affected. A portion of the liver was digested for some time in boiling water, but did not appear to be much affected by it; the water was rendered slightly turbid, and the turbidness was increased by the addition of the bichloride of mercury.

"2. A portion of the liver cut into thin shreds, was strongly pressed between folds of bibulous paper, being at the same time slightly heated; when a very perceptible greasy stain was left on the paper.

"3. A portion of the liver was digested for some time in boiling alcohol; it did not appear much affected. The alcohol upon cooling became turbid, and gradually deposited a small quantity of a white substance; the addition of water threw down a white precipitate in moderate quantity.

"4. A portion of the liver was boiled in sulphuric ether: as the fluid cooled, a film was formed on its surface; and on the addition of water, a white precipitate fell down.

"5. The portions of liver that had been heated either in alcohol or in ether, or even in water, when dried by exposure to the atmosphere were much reduced in bulk, and were converted into a dark-coloured, hard, dense, partially friable substance. When broken into fragments it was easy to discern the white part, in the form of small rounded masses of various sizes, from the head of a large pin downwards, and of a somewhat shining appearance; the whole being intersected in various directions by membranous bands.

"6. Some thin slices were macerated in a solution of caustic potash at the temperature of the atmosphere; the red part appeared to be much more acted upon than the white matter, which was scarcely if at all affected. The slices were then suspended in water, when a white sediment was gradually formed, while the water presented the appearance of an imperfect solution of soap; it became more turbid on being boiled, and acetic acid threw down a considerable precipitate from it.

"7 A portion of the liver was heated in a solution of caustic potash; the texture was completely broken down, and a substance rose to the surface, which, when removed, was found to be of a thick consistence, unctuous, and inflammable, leaving after combustion a considerable portion of a light carbonaceous residuum. It was not melted by the heat of boiling water, but when held over the flame of a spirit-lamp on a leaf of platina, it immediately fused. Acetic acid threw down a gray precipitate from the potash.

"8. A portion of the liver was digested in diluted nitric acid at the temperature of the atmosphere. In six days its texture was entirely broken down, the red matter appeared to be dissolved, while the white substance was left in the form of small grains. These grains were separated from the acid and washed in water; they were found to be fusible at a temperature above that of boiling water, and were readily inflamed. A portion of the acid was supersaturated with ammonia; it assumed a deep orange colour, but no precipitate was thrown down.

"9. The substance which was procured by the action of heated alcohol on the liver was of a thick and somewhat unctuous consistence; it was softened but not fused by the heat of boiling water; but when exposed to a stronger heat on bibulous paper, it fused and left a greasy stain. It immediately melted by being heated over a spirit-lamp on a sheet of platina, and burned with a bright flame. When digested with pure ammonia it partially united with it and formed a saponaceous substance. It was scarcely acted on by being heated with caustic potash, but by the addition of acetic acid to the potash after it had been filtered, a slight precipitate was thrown down.

"From the above observations I think we are warranted in concluding, that the liver which you sent me for examination contained a quantity of a substance nearly resembling cholesterine, the body which forms the basis of the biliary calculi. I do not venture to determine concerning the nature of the connexion which subsisted between this substance and the liver, but I should conjecture that it had been secreted by the arteries of this organ, and deposited in its cellular texture.

Believe me, dear Sir, most truly yours,

J. Bostock."

The general conclusion drawn by Dr. Bright, with regard to the liver is, that there are three distinct conditions of disease in that viscus which may be connected with dropsy.

1.—A distinct morbid deposit, as in the case already given.

2.—Both the secreting part and the connecting cellular tissue may suffer a change of structure nearly in an equal degree, the whole viscus becoming unusually hard; the acini becoming enlarged, and the parenchymatous substance assuming a cartilaginous firmness, but without being drawn into bands. In other instances, however, bands are formed not unlike true schirrus.

3.—The whole organ may be changed into globular concretions, harder and tougher than natural, and which are easily picked out of the cavities in which they are embedded. In a case where a portion of liver thus diseased was macerated for some months in water, the effect was to convert all these globular bodies into adipocere, the connecting cellular membrane undergoing no corresponding alteration.

The attention is next directed to some diseased conditions of the heart, and great vessels producing dropsy; but nothing very interesting struck us on the perusal.

The different results of inflammation attacking different textures of the lungs are next considered, and the principal object of our author appears to be to establish a position so familiar to us, that we can scarcely suppose it is not generally admitted, we mean that such a difference does actually exist, and may be recognised by the symptoms during life, and by the appearances after death.

The cases first detailed are various forms of bronchitis, a disease which the author justly observes is altogether distinct from inflammation of the substance of the lungs, and requires very different treatment. We repeat, however, that we believe the author is mistaken if he supposes that the opinion which he advances on this subject is at all peculiar or different from that generally entertained.

Under the head of termination of pneumonia in suppuration, there is a very good case of simple abscess of the lungs; but in most cases where suppuration takes place from the violence of simple inflammation, it is attended by sloughing.

Gangrene of the lungs is known to be a very uncommon termination of inflammation in these organs; probably, however, it may occur more frequently than has been suspected. Several cases are described by Dr. Bright, in which gangrene was found to have taken place.—Some of these were accompanied by intolerable fetor of the breath, and appear to have resembled very much those cases described by Dr. Chambers, in this Journal for September, as examples of abscess with sloughing. The subject is much more fully entered into by Dr. Chambers in the paper alluded to, than by the author before us. The disorganizations of the lungs comprehended under the general appellation of phthisis, are described with much care.

"The history of Phthisis Pulmonalis is unfortunately so familiar to every practitioner, that it is unnecessary to enter into a detailed narrative of symptoms. I shall satisfy myself with little more than a statement of the morbid appearances presented, when, as is most frequent, the disease has terminated fatally; and for this purpose the cases will be so selected as to comprise the greater part of the organic changes which usually take place. These changes will be found chiefly to consist in the disorganization of the lungs, in the ulceration of the larynx, in the obstruction of the absorbent glands, and in the irritation or ulceration of the mucous lining of the intestines. Of these it may be said that the first only is essential to the disease; but all the others are found very frequent to exist, aggravating the symptoms and hastening the fatal termination. The disorganization of the lungs, which more particularly belongs to phthisis, is generally considered to be peculiar to the disease, and to differ specifically from the disorganization which depends on simple inflammation under any of its forms: it is however not to be doubted, that ordinary inflammation has frequently been the immediate forerunner and even the exciting cause of phthisis, giving occasion to that morbid action which shows itself by the formation of the genuine phthisical tubercle. It will be seen in the following cases, that the tubercular deposit assumes two totally different forms; so that, were it not for the fact of their occurring so frequently together in the same individual, in the same lung, and even in the same lobe of that lung, we should scarcely be authorized in considering them the result of the same, or even of analogous morbid actions. It will be seen that sometimes a portion of the lung, varying from the size of a nutmeg to the greater part of a whole lobe, has become of a dense semicartilaginous consistence, has assumed a permanent blueish gray colour, and is nearly translucent; that this has gone on gradually to become yellow in spots, to soften, and to form abscesses of a sluggish character, finding their way in time to some unobstructed bronchus, and discharging themselves by expectoration. The cavity becomes larger and larger by the suppuration of the internal surface, which appears like a secretion from a vascular tissue, with which it is surrounded; this however is not exactly the case, as there is reason to think that the cavity actually enlarges, and therefore that the internal surface must waste, and be renewed by a fresh membrane forming beneath. In other parts of the same lung a very different process has often been going on; and a number of minute bodies, not larger than the smallest shot, have been deposited more or less thickly throughout the substance. The lung may scarcely be altered in its appearance; but, on pressing it, hard unyielding bodies are distinctly felt; these in a short time enlarge, and when still not larger than small peas, begin to suppurate at the centre; or more frequently these small miliary tubercles join in clusters, either forming masses of themselves, or inclosing a piece of the lung, which becomes hard and blue and semi-transparent, and either runs into suppuration together with the small tubercles, or, if no suppuration takes place towards the centre, becomes completely separated, and forms a slough in the midst of the cavity, which is surrounded by the tubercular matter in the form of a cyst. These are the two extreme points of the processes which take place in genuine tubercular phthisis; and they admit of many modifications, more particularly when combined with the effusion of fibrin which attends simple inflammation.

"Another of the morbid changes frequently attending phthisis is the ulceration which takes place about the larynx and the trachea. It is often very difficult to say whether this or the affection of the lung has first existed; there is no doubt that sometimes the one and sometimes the other precedes. The occurrence of this ulceration is generally betrayed by the hoarseness of the voice and the clanging sound which accompanies the cough; the most usual seat of the ulceration is immediately below the *rima glottidis*, where it begins with one or two very small round ulcers, which soon extend and become irregular in form, assuming the appearance of superficial abrasion: the situation and extent, however, vary a little; sometimes the epiglottis itself is ulcerated, and occasionally small independent ulcers take place in the mucous membrane of the trachea, two or three inches below the cartilages of the larynx. Where the ulceration of the larynx has taken place early, it has not unfrequently drawn the attention both of the patient and practitioner from the more important seat of disease; for the irritation and uneasiness occasioned by this local disease in the orifice of the air-passage, is much more forced upon the attention, than the inconvenience and dyspnœa, seldom amounting to pain, which accompany the tubercular deposit in the lungs.

"A third set of morbid appearances in phthisis are those which arise from the obstruction of the absorbent glands, more particularly those of the bronchial passages and of the mesentary. In scrophulous subjects we often find the glandular system very generally disordered in connection with phthisis; but in other cases, where little or no glandular disease is discovered in parts which are open to observation, the glands of the mesentery are still enlarged and hardened, or have gone into a state of suppuration. This disease of the mesenteric glands appears frequently to be the immediate consequence of the irritation taking place on the mucous coat of the intestines; which often exists from an early period of the complaint, showing itself at first by irregular action of the bowels, by occasional diarrhœa, by the natural cleanness of the tongue, its glossy appearance, or the occurrence of apththæ; and in the latter stages by the uncontrollable diarrhœa which hastens the conclusion of the disease. It will generally be found that the enlargement of the mesenteric glands in any one part maintains a certain proportion to the degree of irritation in the mucous membrane of the intestine immediately opposite; and that when ulceration has actually taken place, the mesenteric glands are largest and most inclined to suppurate in the immediate neighbourhood of the ulcers. When the glands of the mesentary become obstructed, a fresh cause of that rapid emaciation which attends phthisis unfolds itself, and the careful examination of the part after death often explains that cause in a most striking manner. It appears that the condensed structure of the gland actually presents a mechanical obstruction to the fluid which has been separated for the nourishment of the body; and I have found the lacteals gorged with chyle, as by the most successful injection. This is seldom seen to any great extent: but very frequently occurs partially, so that a few branches of the lacteals are beautifully traced, filled with a fluid which separates, throwing up a cream-like substance to the top, while its more watery portions remain in the lower part of the fine tubes: and by carefully following these tubes, you may sometimes trace two or three of their convolutions in the substance of the gland to which they run. At one time I was inclined to think that the lacteals were seldom found to be obstructed, except when they originated from an ulcered surface in the intestines, but this is not the case; and though, owing to the greater irritation which is set up in these parts, the glands will generally be more diseased and hence less pervious, and therefore will be most likely to give occasion to the filling of the lacteals; yet I have sometimes traced the gorged lacteal most distinctly, from a part of the mucous membrane free from ulceration.

"The bronchial glands also, as I have before remarked, are subject to morbid affections in phthisical subjects. They occasionally become remarkably dark in their colour, firm in their consistence, and enlarged to a very unusual degree; sometimes, though rarely, going into suppuration, but much more frequently becoming the seat of an earthy deposit towards their centre.

"The last seat of morbid change of which I shall speak, is the mucous membrane of the intestines. The affection of this membrane is one of the most important features in a majority of the cases of phthisis; it shows itself by unequivocal symptoms during life, and is traced in two different forms after death; sometimes giving proof of a diffused irritation along the whole membrane from the pyrolous to the termination of the rectum, evinced by increased vascularity or by the appearance of innumerable minute black specks, which give a general gray colour to all the parts where they are most frequent, and sometimes affording evidence of a more severe affection, by the formation of numerous ulcers, which are found sometimes in the upper part of the duodenum, frequently dispersed along the whole course of the intestines, but usually most abundant about the valve and through the whole extent of the colon. These ulcers, as found in the small intestines, are usually in the first place very small and circular, and appear to originate from round opake white bodies about the size of half a sweet-pea; but whether these are altogether morbid tubercular deposits, or are only enlarged mucous glands, it is no easy matter to decide: certain it is, that they are most generally placed in that part of the circumference of the intestine which is most distant from the mesentary, and where the mucous follicular structure is most developed. These ulcers often extend to the size of a shilling, and sometimes still further, in a lengthened form. It is remarkable that the edges are often thickened by an opake white matter deposited beneath the membrane and drawing it into a puckered form; and sometimes this deposit is seen in the middle of the ulcer, like a granulation on its surface. The ulceration of the large intestines is usually the most remarkable, and it varies a good deal in its character ; in general it is by far the most conspicuous about the cœcum and the valve of the colon, where it appears to commence much as in the small intestines, by opake deposits ; but these are from the beginning more numerous, and it goes on to a much greater extent, sometimes involving the cœcum in one continued ulcer ; this occasionally, though rarely, affecting the lining membrane of the vermiform process itself. In other parts of the colon, as along its arch, the ulceration usually assumes a more regular and uniform character that in the cœcum ; for we generally find a series of oval ulcers with elevated edges, more or less closely distributed along the sides of the longitudinal bands, and apparently determined in the direction which they take, by the transverse folds of the mucous membrane. These ulcers frequently continue to the sigmoid flexture, and occasionally even to the rectum. It appears to me that they sometimes undergo a process of healing, their tubercular edges softening down, and their flattened edges adhering to the parts which have been denuded by the ulceration ; but this is not a frequent occurrence, because the more usual course of phthisis is to go on from worse to worse till it terminates in death ; and little attempt has usually been made by practitioners to change the condition of the intestines, while they have felt that the more urgent disease was in another part." (P. 148.)

In one of the cases of phthisis, which does not appear to have presented any thing remarkable in the symptoms, the aorta and some of the arteries were found to be of a bright red internally ; we remember to have heard the late Mr. Shaw state, that he had frequently seen this in the bodies of persons brought to the dissecting-room, who had died of phthisis pulmonalis.

Two cases follow, which are intended to show the effects of Ipecacuanha and mild Mercurials in dysentery: we believe the fact will be readily admitted.

The rest of the volume is occupied with an account of the morbid appearances which present themselves in the intestines during the progress of fever, and on the method of treatment to be adopted. The readers of this Journal are, or ought to be, well acquainted with this subject already, as it has been fully discussed in the papers of Dr. Chambers and Dr. Hewett. The observations of Dr. Bright, both as to pathology and practice, are confirmatory of those already made by these gentlemen, and the accordance between physicians to different hospitals thus manifested gives additional strength to our belief of their accuracy. Did Dr. Bright's views differ in any important point, we should lay them before our readers and contrast them with the others, but such not being the case, we consider that to enter upon the subject further would be a work of supererogation.

The pathological points are illustrated with fifteen highly finished engravings. Some of them are extremely good, but others are comparatively inferior; and, upon the whole, we think the colouring exaggerated. They are by no means so good as the plates in Dr. Hooper's work on the brain, although the volume is proportionally more expensive.

---

*A Treatise on the Cutaneous Diseases incidental to Childhood: comprehending their Origin, Nature, Treatment, and Prevention.* By WALTER C. DENDY, Surgeon to the Royal Infirmary for Children, &c.—8vo. pp. 289. Churchill, London, 1827.

THE errors of the ancient writers upon the subject of cutaneous affections have been so very frequently the theme of modern criticism, that it cannot be necessary we should (to adopt the language of Mr. DENDY,) "turn the sail of our investigation up the stream of time!" in order to discover the precise extent of their knowledge. It is confessed that the terms formerly employed to designate diseases of the skin were very loosely and indiscriminately applied, and that, in consequence, the treatment adopted was equally deficient and inappropriate.

If, however, the labours of the learned of olden times effected but little in this branch of medical science, it has received considerable improvement at the hands of different modern authors, whose names and works are too well known to require any formal eulogy from us.

The arrangement of Mr. PLUMBE, as well as the classification lately presented to us by Mr. RAYER, are considered by the author "as very correct elucidations of the character of cutaneous diseases; but, as the nosological arrangement should, in my opinion, present an epitome, as it were, of the Treatise, I think the combination, for example, of all cutaneous inflammations, whether acute or chronic, essential or symptomatic, in one chapter, by Mr. Rayer, must occasion some confusion with regard to the modes of treatment; while, in Mr. Plumbe's arrangement, certain diseases are unnecessarily disjoined." (P. 6.)

Mr. Dendy deals more mercifully with ALIBERT than the late Dr. BATEMAN, who declared that the French writer, with all his boasting, "had contributed nothing to the elucidation of the obscurity" in which the subject had been previously veiled. Mr. D. confesses that Alibert stands high in the rank of writers on cutaneous diseases, but that, in consequence of his imperfect classification, his brilliant work must "moult some feather of its value."

Mr. Dendy almost implicitly adheres to the phraseology of Dr. Willan.

The author has more than once regretted the *local* success of his applications to the crusts of porrigo, the removal of which has been speedily followed by acute ophthalmia, which without great care would have proceeded to the rapid disorganisation of the globe; and he presumes we may often trace the occurrence of severe cerebral affections to the merely local treatment of porriginous or impetiginous crusts. We have more than once had occasion to touch upon this question. It has never fallen to our lot to lament the success of our local treatment in cutaneous affections, provided the bowels were acted upon by occasional purgatives at the period of their subsidence.

It is true that Dr. Darwin has presented us with two cases of chorea, and one of hepatic disease, immediately succeeding the cure of scabies, and that a few other similar instances have been recorded. But when we consider the immense number of cases of that, and many other eruptive diseases, which are cured by the popular application of local means, and the very few examples of subsequent and connected constitutional injury, we are inclined to be a little doubtful of the fact. We, perhaps, too frequently argue upon the principle of *post hoc ergo propter hoc;* the fallacy of which sophism the author deprecates upon the subject of the virtues ascribed to many remedies. The proof of the danger above alluded to would be the frequent occurrence of constitutional disease after the removal of cutaneous affections. We should not, ourselves, allow the few cases of the above kind which have been related to have much weight in our practice. We should require very unequivocal evidence of the connexion between the removal of the disease of the skin, and any subsequent general malady which may occur. It must be granted, "that from the recession of the specific exanthemata, as rubeola and scarlatina, we may usually anticipate an aggravation of the febrile symptoms," and sometimes even dangerous visceral diseases; and we perfectly agree with Mr. Dendy, "that the administration of internal remedies is almost universally essential in the treatment of cutaneous diseases: indeed, indispensable in those of children."

The author has been anxious to dwell much on infantile expression, "as infantile disease has been too much consigned to the often superstitious and bigotted priestess of the nursery, from an absurd idea that it was useless to attempt to investigate disease where words were wanting to develop the sufferings of the patient." We were really not aware that the profession were exposed to such an accusation of negligence and folly.

The author justly presumes, that, with but a limited list of remedies, we may affect more, with an attentive consideration of constitution and the *cause* of disease, than with the whole materia medica at our disposal, assisted only by a superficial and confined view of the malady with which we have to contend.

From the conviction that the majority of infantine eruptions are symptomatic of constitutional or intestional disease, Mr. Dendy has deemed it proper to offer some remarks on the establishment of the healthy condition of the functions, which may be considered as constituting the prevention of cutaneous diseases. In this section we find many judicious practical observations, which have, however, been much too frequently noticed to require any particular comment.

The error of feeding children to excess would rarely be committed if the advice of the medical world had its proper influence on the public. But, in spite of all our rhetoric and remonstrance, mothers will still be found who will persist in *stuffing* their children, or, as Mr. Dendy expresses it, who appreciate them "as they would their capons or their pigs, by the mass of matter which composes them."

We enter upon the more immediate subject of the volume, by the consideration of those cutaneous diseases symptomatic of disorder in the alimentary viscera. Mr. Dendy observes, that the papulæ of strophulus "are often materially modified by the peculiarity of constitution and mode of living: indeed, by these causes, and by neglect, it may be totally changed from its first simple form to one even of contagious nature. From this source arise the peculiarly aggravated cases of eruption among the children of the indigent, in whom the simple strophulus will sometimes degenerate into the contagious scabies." (P. 80.)

We suspect there is some error in this opinion. From bad feeding and disordered secretions, that common and generally trifling disease, strophulus, may, we know, be much exasperated. There may exist a considerable degree of itching, pain, and excoriation; but we should still doubt the contagious nature of the disease, however aggravated it may become from accidental circumstances.

A very brief account is given of prurigo, crusta lactea, follicular tumors, phlegmonous tumors, urticaria, impetigo, and herpes; and we then arrive at "Disease, symptomatic chiefly of deranged chylopoietic functions, usually marked by debility." Mr. Dendy is of opinion that purpura hæmorrhagica is usually dependent upon a debilitated and deranged state of the system. Some pathologists have referred the ecchymosis to increased vascular action, which they have termed the hemorrhagic effort. We believe our author has formed a correct view of the subject, although cases may occasionally occur characterised by such symptoms as to support the contrary doctrine. Mr. Dendy argues, that—

"From the absence of acute fever, the condition of the pulse, and the languid state of the system, inducing often syncope on exertion, occurring, however, without previous excitement, it may be termed a disease of debility, depending on a want of the elastic property of vascular coats, allowing the blood to break through the capillaries: a doctrine in opposition to the opinion of Dr. Parry, who asserted its inflammatory nature. In what is designated petechial fever, however, which I have never seen in children, the vascular action may be much increased. On the first effusion of the blood, it is often of a florid hue, but it speedily becomes purple, assuming a brownish tinge as it is about to be removed by absorption. The cuticle above the spots is slightly elevated and smooth; sometimes, however, it is raised in vesicles filled with purple blood. In purpura hemorrhagica, as well as scorbutus, the mucous surfaces are often more affected than the external skin, the danger being then materially increased, as the delicate epithelium is easily burst by the extravasated blood, and thus we have hemorrhages from the gums, the throat, &c. which have given the specific name to this form. Internal hemorrhage may thus take place, which sometimes prove fatal. It is rare that hemorrhage takes place on the surface of the body, but in some cases the slightest pressure will produce ecchymosis, and firm compression will break the cuticle, and the blood will flow from the lesion. I have seen children evince this petechial tendency, being spotted black by the slightest bruises. The extravasation in very extreme cases will sometimes occur from the mere muscular action of a limb. Blood will sometimes flow from the eyes, nose, and mouth of children, at the same time that they expectorate bloody mucus, and pass bloody stools resembling melæna, consisting only of sanious coagula mixed with intestinal mucus. These appearances usually occur in children about the fourth or fifth year. In protracted cases, where internal hemorrhage has not speedily proved fatal, we see the blood gradually become paler, until at last the hemorrhage will scarcely stain the linen on which it drops: the ecchymosed spots being subject to the same faded change; the circulating fluid appearing to assume almost the character of serum. The cases which generally prove rapidly fatal, are those in which hæmatemesis, hæmaturia, or hæmoptoe occur, the effusion being both obscure and out of the reach of any mechanical pressure. In similar cases the heart and liver have been found very pale in their internal fibrous or parenchymatous structure, a livid colour appearing under their internal membrane." (P. 143.)

For the removal of this species of purpura, we must chiefly rely upon the judicious use of mercurial purgatives. Some caution, however, is required. "Mercury should not be continued long in purpura, as it has usually a tendency to depress power, although some physicians recommend it even to ptyalism."

The author next speaks of "diseases usually symptomatic, arising also from extraneous excitement; depending probably on peculiar idiosyncrasy." As in the former section, we pass over the descriptions given of many different species of cutaneous disease, because we find them to be merely superficial abstracts of opinions and statements that have frequently been repeated. We differ from Mr. Dendy upon the treatment of porrigo favosa, which he states, "even in those cases which arise from actual inoculation, should be chiefly internal." That internal remedies are necessary in this very obstinate form of skin-disease, we readily admit; but we know, also, that quite as much depends upon the local applications, of which the best are (as stated by the author) poultices at the commencement of the disease, and, on the subsidence of inflammatory action, the Ungt. Picis p. j. Ungt. Sulph. p. 2. In some very stubborn cases, Mr. D. has found equal parts of nitric acid, oil, and water, very efficacious.

Upon the subject of porrigo suctulata, we agree with Dr. Underwood and Mr. Plumbe. We do not remember a single case, out of very many we have had under our care, in which the disease of the surface appeared to be connected with constitutional derangement. Mr. Dendy thinks otherwise, and assigns a double origin to this complaint. The following ointment has been found very efficacious in this disease:*

℞. Sod. Alicant. ℨiij.; Potass. Sulph. ℨiij.; Adip. ℥iij. M.

It is to be applied daily after the employment of poultices. A decoction of tobacco has been used as an external remedy, but this must be employed with caution, as death has occurred from its injudicious use.

* Formulaire Pratique des Hôpitaux Civils de Paris.

We cannot pass over the subject of "fungous excrescence" round the root of the nail, without expressing our approbation of the manual dexterity shown by Mr. Durlachre, in his excellent operation for the removal of this disease. We have twice seen Mr. D. operate, and in neither case did the patient complain or appear to suffer pain. It must be needless to observe, that the rude and unscientific operation of tearing away the nail, as it is even now sometimes practised, inflicts the most severe torture. Mr. D.'s operation "consists in cautiously cutting through the nail, with a very small knife, and without dividing or wounding the cuticle interposed between its under surface and the sensitive structure beneath it; then, with a minute pair of forceps, raising and detaching the nail. The operation is productive of little or no pain, and is eminently successful."

"Diseases consequent to specific infection," come next.—We have but one remark to offer upon the sketch which the author has given of Rubeola. It is stated to occur but once during life. To this general fact, however, there are occasional exceptions:—we have ourselves, in two instances, seen measles a second time in the same subject; and numerous cases were published by the late Dr. Baillie, the accuracy of which there can be no doubt. Mr. Dendy has not himself made any experiments upon the inoculation of measles, "but it has been advocated by some pathologists, and, in the Bibliotheca Italiana, the following proof of its possibility is related: 'The inoculation of measles, which has been already practised with success by Horne and Horst, was repeated by Professor Speranza during an epidemic which reigned at Mantua in 1822. He inoculated six children as well as himself, and the measles in each case appeared in a mild and regular form. A slight incision was made in the best-looking measle, and, in the blood which flowed from the scratch, the point of a lancet was dipped, which was then inserted into the arm.'—A Mr. Fortual has stated in a German Journal, that all the children to whom he administered sulphur were protected from measles, at a time when this disease was epidemic. How far this was coinciding, or there was really a prophylactic effect in the sulphur, I cannot determine. This formula was:—R. Sulph. ℥ss.; Sacch. a. ʒj.; Coch. nim. ss. bis terve indies." (P. 218.)

We could not dwell upon the remaining pages of this volume with satisfaction to ourselves, or advantage to our readers. They contain short practical accounts of several of the cutaneous affections incidental to childhood.

We cannot offer a very favorable opinion of Mr. Dendy's performance. There are indeed some trifling changes in the arrangement and verbal description of the various maladies touched upon, which are just sufficient to rescue the author from the charge of having copied his matter from others who have before published upon the same subject. There was certainly no vacant niche in the professional library for the book Mr. Dendy has presented to us. The substance of it may be found in most works which treat of the diseases of infants; and, in either Bateman or Plumbe, we have much more satisfactory descriptions of the cutaneous diseases which are here rather superficially considered. The duty of reviewing is never more unpleasing to us than when we are compelled to withhold our praise; but, from misplaced commendations, the conclusion is *non melius de laudato, pejus de laudante.*

---

*Introduction to the Science of the Pulse, as applied to the Practice of Medicine.* By JULIUS RUCCO, M.D. Bachelor of Medicine in the Royal University of Naples; ex-Professor of Anatomy and Comparative Physiology in the Royal Medico-Chirurgical College, and Member of the Royal Institute of Encouragement of that Capital; Licentiate Member of the Royal College of Physicians in London; ordinary Member of the Class of Natural Sciences of the Italian Academy of Sciences, Letters, and Arts; Corresponding Member of various Medical and Literary Societies of Europe and the United States of America; Author of various Medical Works, &c. In two Volumes.—Large 8vo. pp. 353, 452. Longman and Co. London, 1827.

EIGHT-hundred-and-five royal octavo pages on the pulse!—the work should have been written last century. We sat down to the perusal with every disposition to be pleased, and every wish to be instructed: we had heard the author favourably spoken of, and knowing him to be a foreigner, we were desirous of showing him courtesy. But we have a higher duty to perform—we must be candid. In our judgment, then, the work is very much laboured—very speculative—very learned—and very useless.

Dr. RUCCO directed his attention to the pulse so early as the year 1804, when he held the office of Regius Professor of Pathology at Naples. He connected himself with many learned men of Italy, who "contributed to dissipate the thick darkness which obscured the horizon of medical knowledge," and, by their co-operation and assistance, advanced the study of '*Sphygmica,*' a science through which nature speaks to the physician, by means of its organ—the pulse.

In 1811 our author was sent by his government to Paris, where he appears to have remained four years, prosecuting his medical researches in that capital. In 1814, he returned to

Naples, having been appointed Professor of Anatomy and Comparative Physiology. This new situation distracted his attention from his favourite study; but this apostacy was of short duration, as in 1815 he was seized with a desire of examining the phenomena of disease in different climates; "an idea which, singular as it may appear, finally determined him to sail for the United States towards the close of 1815, thus incurring all the disadvantages of a voluntary exile." He settled at Philadelphia till 1820, when he came to London "for the purpose of the still further prosecution of his researches."

The short sketch of his history with which he has presented us, will show how much the author has been in earnest, and what sacrifices he has made in the progress of his object. It has been supposed, indeed, that circumstances of a political nature had led to his abandoning his native country; but, as he expressly states his exile to be "voluntary," this idea must be unfounded, although it certainly would have accounted more rationally for his giving up the anatomical chair at Naples, and wandering over the face of the earth in the manner he appears to have done. Neither does Dr. Rucco know the feelings of those among whom he now resides, if he supposes they would have thought the worse of any man who had retreated from the tyranny with which Austria rules his degraded, but still noble, country.

After a long introduction, in which the author gives the history of the Spygmical art, we have a succession of elaborate articles on the Anatomy and Physiology of the Heart and Arteries; and these are followed by a chapter on the "Exploratory Organ of the Pulse;"—which expression means neither more nor less than the fingers. The anatomy of the exploratory organ is given with tedious minuteness; and this fact may serve as an illustration of those frequent digressions from the proper object of the work, which render it laborious in perusal, and most difficult to digest. Another circumstance, and we must say another blemish, is the unnecessary repetition of words, which swell the size of the work, already too great. As an illustration of our meaning, take the following, in which we have put the repetitions in italics.

"That man's natural pulse varies in proportion to the changes which take place in his organization, is an indubitable truth, the evidence of which is supported by the testimony of nature herself, when we observe that the pulse of man, without losing an atom of its natural character, can indicate the state of health in three individuals of different ages, although it may be *quick, frequent, and*

*small* in the first; *elevated, full, and strong* in the second; and *slow, rare, and hard* in the third. The pulse is, in fact, *quick, frequent, and small* in children; *elevated, full, and strong* in adults; and *slow, rare, and hard* in old persons. But it is not sufficient to know, or to say, that the pulse is *quick, frequent, and small* in children; *elevated, full, and strong* in adult age; and *slow, rare, and hard* in old persons: to this information it is requisite to add the knowledge of the fact, that the *celerity, frequency, and smallness* of the pulse in the period of infancy vary in degree and strength; that the same takes place with respect to its *elevation, fulness, and strength* in adult, and its *slowless, rarity, and hardness* in old age." (Vol. i. p. 151.)

The second volume is that from which we expected to derive most information, as it relates to the Morbid Conditions of the Pulse. These are divided into the diagnostic, organic, and critical.

The diagnostic pulses are successively treated of under the heads of the "Great Morbose Pulse;" and the "Small Morbose Pulse;" and a general idea of the author's opinions may be gathered from the following analysis of the inferences drawn from different kinds of pulse.

"1st. If the pulse, from being small, becomes suddenly great, without the strength of the patient being improved, it is a sign of death in lethargy and apoplexy, according to the observations of Baglivi and Wapfer.

"2d. The change of the rare and slow pulse into quick and frequent, is a favorable sign in ataxic fevers and hyposthenic diseases.

"3d. The softness and rarity which succeed the excessive hardness and frequency of the pulse in acute hyposthenic fevers, is an excellent sign.

"4th. According to the observation of Actuarius, the elevated, vehement, and quick pulse announces the commencement of a disease in one or more vital organs; and the small, frequent, and weak pulse indicates, on the contrary, the long duration of a disorder, which finally succeeds in impoverishing and overpowering the vital powers.

"5th. The thinness, frequency, and hardness of the pulse show the state of irritation, or crudity, as it is called by medical practioners. If the pulse continues thus in the successive periods of acute diseases, after the epocha of the concoction, it is a sign evidently unfavorable; for it is then supposed that the crisis has not taken place there, or that, if it does take place subsequently, it will not be true or salutary.

"6th. The frequent, small, irregular, and thin pulse, whilst it generally indicates the concourse of spasm, irritation, and weakness, is also the worst sign in pestilential fevers, in typhus, in spurious angina, in false peripneumonia, and in epidemic dysenteries.

"7th. The sudden change of the great and full pulse into the frequent, small, thin, and irregular pulse, is a fatal sign in acute diseases.

"8th. The greatness and fulness which succeed to the smallness, weakness, frequency, and irregularity of the pulse, is a sign undoubtedly favorable in acute diseases.

"9th. The pulse which vacillates and changes its form every instant, is always a fatal sign. This vacillation, when it exists, is observed at one time strong, at another time weak, at one time rare, at another frequent, at one time quick, at another slow: when the pulse thus vacillates and changes, it is a sign that the pericardium, or the heart, or even both, are affected by inflammation.

"10th. When the beating of the pulse becomes very sensible throughout the whole of the arterial tree, in the presence of acute diseases, it is an unfavorable sign, because such a phenomenon arises from the deep and general inflammation which is precisely indicated by this sensible beating throughout the whole arterial tree.

"11th. The frequency, weakness, and inequality of the pulse is an unfavorable sign, as it indicates the organic affections of the breast, according to Hoffmann's observations.

"12th. The smallness, rarity, and weakness (approaching to insensibility) of the pulse, is an unfavorable sign, especially in syncope.

"13th. The pulse which grows weaker, proceeding from the greatness to the insensibility of its oscillations, is the worst sign, as it indicates the successive exhaustion of vitality." (Vol. ii. p. 104.)

The organic pulses are those which, by some peculiarity of character, point out the particular organ which is diseased. The author evidently feels this to be a weak point, as he declares his intention not to notice those who assert "that the doctrine of organic pulses is nothing but the produce of imagination." We have an organic pulse for the head, the throat, the chest, and—the nose; after which follow those for the abdominal and pelvic viscera. We were rather sceptical as to the nose, but it is quite obvious that our author has no doubt about the matter, as will appear from the following passage, which at the same time will serve to show the vagueness and unsatisfactory nature of the whole hypothesis.

"In fact, the existence of the nasal organic pulse is certain in the presence of local affections of the nostrils or of the nose. The pulsatile artery generally loses its cylindrical form; for, whilst its posterior and middle or central part, which correspond to the point of the little, annular, and middle fingers, rises in the form of a small hillock: its anterior, or digital part, on the contrary, appears to flatten itself, like a small nervous riband, under the tip of the index finger: thus, it is in this track of the flattened artery that we perceive in motion certain round bodies, similar to so many drops of water, which come driven with force against the apophysis of the radius, and from which the shock appears to be transmitted to other drops which follow the first. At other times a kind of formiculation is felt, or otherwise the impression of a movement of only two round and large bodies, which run rapidly against the index finger."

Of the critical pulses, we shall say nothing, being infidels on the subject of crises in general.

We regret much that we can say nothing very favorable of Dr. Rucco's work; for it must have been attended with prodigious labour, and the sacrifices he describes himself as having made in the prosecution of his object have been very great. He appears deeply read in the older medical writers, and makes constant reference, among others, to GALEN, whose authority he seems to look up to with great reverence. But the admiration of Galen is not quite so great among us, and we remember, on a recent occasion, to have heard a public lecturer, who, desirous of illustrating the absurdity of wild hypothesis—of substituting words for ideas, gave a quotation from Galen as affording the most appropriate example.

A work on the Pulse certainly might be made very useful; but then it ought to be a simple detail of the signs it affords, without any attempt to carry these to a degree of refinement, which is plausible perhaps in the closet, but wholly inapplicable at the bedside of the patient.

---

*Library of Useful Knowledge. Animal Mechanics.* No. 9. Published under the superintendence of the Society for the Diffusion of Useful Knowledge.—8vo. pp. 31. Baldwin and Co. London, 1827.

THE number before us is the first one of this work which has afforded us an opportunity of introducing it to our readers. It is intended to shew the perfection of design in the bones of the head, spine, and chest, shown by comparison with architectural and mechanical contrivances, and the illustrations are of the most interesting description. We have often heard them in the course of the last ten years in the lectures of a distinguished teacher, who may therefore be presumed to be the author. In our review of Dr. Arnott's work on Physics, we alluded to the excellence of Mr. Charles Bell's oral discourses on Animal Mechanics; and we cannot give our readers a better idea of these, than by making some quotations from the work before us.

After some introductory observations, in which it is pointed out that the contrivances for protecting vital organs are not absolute securities against accident, but that they are calculated to meet all the circumstances of ordinary life, the author proceeds to illustrate the subject, by comparison with some of the many mechanical contrivances of art. Men proceed in a slow course of advancement in architectural, or mechanical, or optical sciences; and, when an improvement is made, it is found that there have been all along examples of it in the animal body, which ought to have been marked before, and which might have suggested to us the improvement. Assuming that the head is the noblest part, the author commences with it, and the manner in which this part of the subject is handled, may be taken as an example of the whole.

"It requires no disquisition to prove that the brain is the most essential organ of the animal system, and being so, we may presume that it must be especially protected. We are now to inquire how this main objected is attained?

"We must first understand that the brain may be hurt, not only by sharp bodies touching and entering it, but by a blow upon the head which shall vibrate through it, without the instrument piercing the skull. Indeed, a blow upon a man's head, by a body which shall cause a vibration through the substance of the brain, may more effectually deprive him of sense and motion than if an axe or a sword penetrated into the substance of the brain itself.

"Supposing that a man's ingenuity were to be exercised in contriving a protection to the brain, he must perceive that if the case were soft, it would be too easily pierced; that if it were of a glassy nature, it would be chipped and cracked; that if it were of a substance like metal, it would ring and vibrate, and communicate the concussion to the brain.

"Further thoughts might suggest, that whilst the case should be made firm to resist a sharp point, the vibrations of that circular case might be prevented by lining it with a softer material; no bell would vibrate with such an incumbrance; the sound would be stopped like the ringing of a glass by the touch of a finger.

"If a soldier's head be covered with a steel cap, the blow of a sword which does not penetrate will yet bring him to the ground by the percussion which extends to the brain; therefore the helmet is lined with leather, and covered with hair; for, although the hair is made an ornament, it is an essential part of the protection: we may see it in the head-piece of the Roman soldier, where all useless ornament being despised as frivolous, was avoided as cumbrous.

"We now perceive why the skull consists of two plates of bone, one external, which is fibrous and tough, and one internal, dense to such a degree that the anatomist calls it *tabula vitrea* (the glassy table.)

"Nobody can suppose this to be accidental. It has just been stated, that the brain may be injured in two ways: a stone or a hammer may break the skull, and the depressed part of the bone injure the brain; whilst, on the other hand, a mallet struck upon the head will, without penetrating, effectually deprive the brain of its functions, by causing a vibration which runs round the skull and extends to every portion of its contents.

"Were the skull, in its perfect or mature state, softer than it is, it would be like the skull of a child; were it harder than we find it is, it would be like that of an old man. In other words, as in the former it would be too easily pierced, so, in the latter, it would vibrate too sharply and produce concussion. The skull of an infant is a single layer of elastic bone; on the approach to manhood it separates into two tables; and in old age it again becomes consolidated. During the active years of man's life the skull is perfect: it then consists of two layers, united by a softer substance; the inner layer is brittle as glass, and calculated to resist anything penetrating; the outer table is tough, to give consistence, and to stifle the vibration which would take place if the whole texture were uniform, and like the inner table.

"The alteration in the substance of the bones, and more particularly in the skull, is marvellously ordered to follow the changes in the mind of the creature, from the heedlessness of childhood to the caution of age, and even the helplessness of superannuation.

"The skull is soft and yielding at birth; during childhood it is elastic, and little liable to injury from concussion; and during youth, and up to the period of maturity, the parts which come in contact with the ground, are thicker, whilst the shock is dispersed towards the sutures (the seams or joinings of the pieces,) which are still loose. But when, with advancing years, something tells us to give up feats of activity, and falls are less frequent, the bones lose that nature which would render concussion harmless, and at length the timidity of age teaches man that his structure is no longer adapted to active life.

"We must understand the necessity of the double layer of the skull, in order to comprehend another very curious contrivance. The sutures are the lines of union of the several bones which form the *cranium*, and surround and protect the brain. These lines of union are called *sutures* (from the Latin word for *sewing*), because they resemble seams. If a workman were to inspect the joining of two of the bones of the cranium, he would admire the minute dove-tailing by which one portion of the bone is inserted into, and surrounded by, the other, whilst that other pushes its processes or juttings out between those of the first in the same manner, and the fibres of the two bones are thus interlaced, as you might interlace your fingers. But when you look to the internal surface, you see nothing of this kind; the bones are here laid simply in contact, and this line by anatomists is called *harmonia*, or harmony: architects use the same term to imply the joining by masonry. Whilst the anatomists are thus curious in names, it is provoking to find them negligent of things more interesting. Having overlooked the reason of the difference in the tables of bone, they are consequently blind to the purpose of this difference of the outward and inward part of a suture.

"Suppose a carpenter employed upon his own material, he would join a box with minute and regular indentions by dovetailing, because he knows the material on which he works, from its softness and toughness, admits of such adjustment of its edges. The processes of the bone shoot into the opposite cavity with an exact resemblance to the foxtail wedge of the carpenter—a kind of tenon and mortice when the pieces are small.

"But if a workman in glass or marble were to inclose some precious thing, he would smooth the surfaces and unite them by cement, because, even if he could succeed in indenting the line of union, he knows that his material would chip off on the slightest vibration.

"The edges of the marble cylinders which form a column are, for the same reason, not permitted to come in contact; thin plates of lead are interposed to prevent the edges, technically termed *arrises*, from chipping off or splitting.

"Now apply this principle to the skull. The outer softer tough table, which is like wood, is indented and dovetailed; the inner glassy table has its edges simply laid in contact. It is mortifying to see a course of bad reasoning obscure this beautiful subject. They say that the bone growing from its centre, and diverging, shoots its fibres betwixt those which come in an opposite direction; thus making one of the most curious provisions of nature a thing of accident. Is it not enough to ask such reasoners, why there is not a suture on the inside as well as on the out?

"The junction of the bones of the head generally being thus exact, and like the most finished piece of cabinet work, let us next inquire, whether there be design or contrivance shown in the manner in which each bone is placed upon another." (P. 3.)

The mechanism of the spine, of the chest, of the bones and joints, of the tendons, and of the muscles, is discussed in the same interesting manner, and the subject illustrated by numerous excellent cuts.

